# Access to community-based eye services in Meru, Kenya: a cross-sectional equity analysis

**DOI:** 10.1186/s12939-024-02244-x

**Published:** 2024-08-26

**Authors:** Luke N Allen, Sarah Karanja, Michael Gichangi, Cosmas Bunywera, Hillary Rono, David Macleod, Min Jung Kim, Malebogo Tlhajoane, Matthew J. Burton, Jacqueline Ramke, Nigel M. Bolster, Andrew Bastawrous

**Affiliations:** 1 Department of Clinical Research, London School of Hygiene & Tropical Medicine, London, WC1E 7HT UK; 2 Kenya Medical Research Institute, Nairobi, Kenya; 3 Ministry of Health, Ophthalmic Services Unit, Nairobi, Kenya; 4 Peek Vision, Kitale, Kenya; 5 Kitale County Hospital, Kitale, Kenya; 6 Moi University, Kipkenyo, Kesses Moi University Road, Kenya; 7 University of Auckland, Auckland, New Zealand; 8 Peek Vision, London, UK

**Keywords:** Equity, Socioeconomic inequalities, Access, Primary care, Primary eye care

## Abstract

**Background:**

Over 80% of blindness in Kenya is due to curable or preventable causes and 7.5 m Kenyans currently need eye services. Embedding sociodemographic data collection into screening programmes could help identify the groups facing systematic barriers to care. We aimed to determine the sociodemographic characteristics that were associated with access among patients diagnosed with an eye problem and referred for treatment in the *Vision Impact Programme,* currently operating in Meru County.

**Method:**

We used an embedded, pragmatic, cross-sectional design. A list of sociodemographic questions was developed with input from key stakeholders. The final question set included the following domains: age, gender, religion, marital status, disability, education, occupation, income, housing, assets, and health insurance. These were integrated into an app that is used to screen, refer, and check-in (register) participants within a major eye screening programme. We gathered data from 4,240 people who screened positive and were referred to their local outreach treatment clinic. We used logistic regression to identify which groups were facing the greatest barriers to accessing care.

**Results:**

A quarter of those screened between April – July 2023 were found to have an eye problem and were referred, however only 46% of these people were able to access care. In our fully adjusted model, at the 0.05 level there were no statistically significant differences in the odds of attendance within the domains of disability, health insurance, housing, income, or religion. Strong evidence (*p* < 0.001) was found of an association between access and age, gender, and occupation; with males, younger adults, and those working in sales, services and manual jobs the least likely to receive care.

**Conclusions:**

Access to essential eye services is low and unequal in Meru, with less than a third of those aged 18–44 receiving the care they need. Future work should explore the specific barriers faced by this group.

**Supplementary Information:**

The online version contains supplementary material available at 10.1186/s12939-024-02244-x.

## Background

More than one billion people currently live with preventable or untreated visual impairment, and over 90% of these cases are easily treatable with highly cost-effective interventions like spectacles and cataract surgery [1]. The vast majority of people with untreated eye conditions live in low- or middle-income countries, and within these countries disadvantaged groups are often disproportionately affected [1, 2]. The ‘central promise’ of the Sustainable Development Agenda is to ‘leave no one behind’, and the World Health Organization (WHO) stress that improving equitable access to care is ‘central’ to Universal Health Coverage (UHC) [3, 4].

Extending equitable access to eye services is a global health priority that has recently been reaffirmed by the historic World Health Assembly Resolution on Vision and the recent Lancet Global Health Commission on Global Eye Health [1, 5]. Identifying and addressing inequitable access to community-based eye services also aligns with the principles of Primary Health Care: an approach to health that prioritises the worst-off and seeks to advance equity and *health for all*[6, 7].

**Fig. 1:**
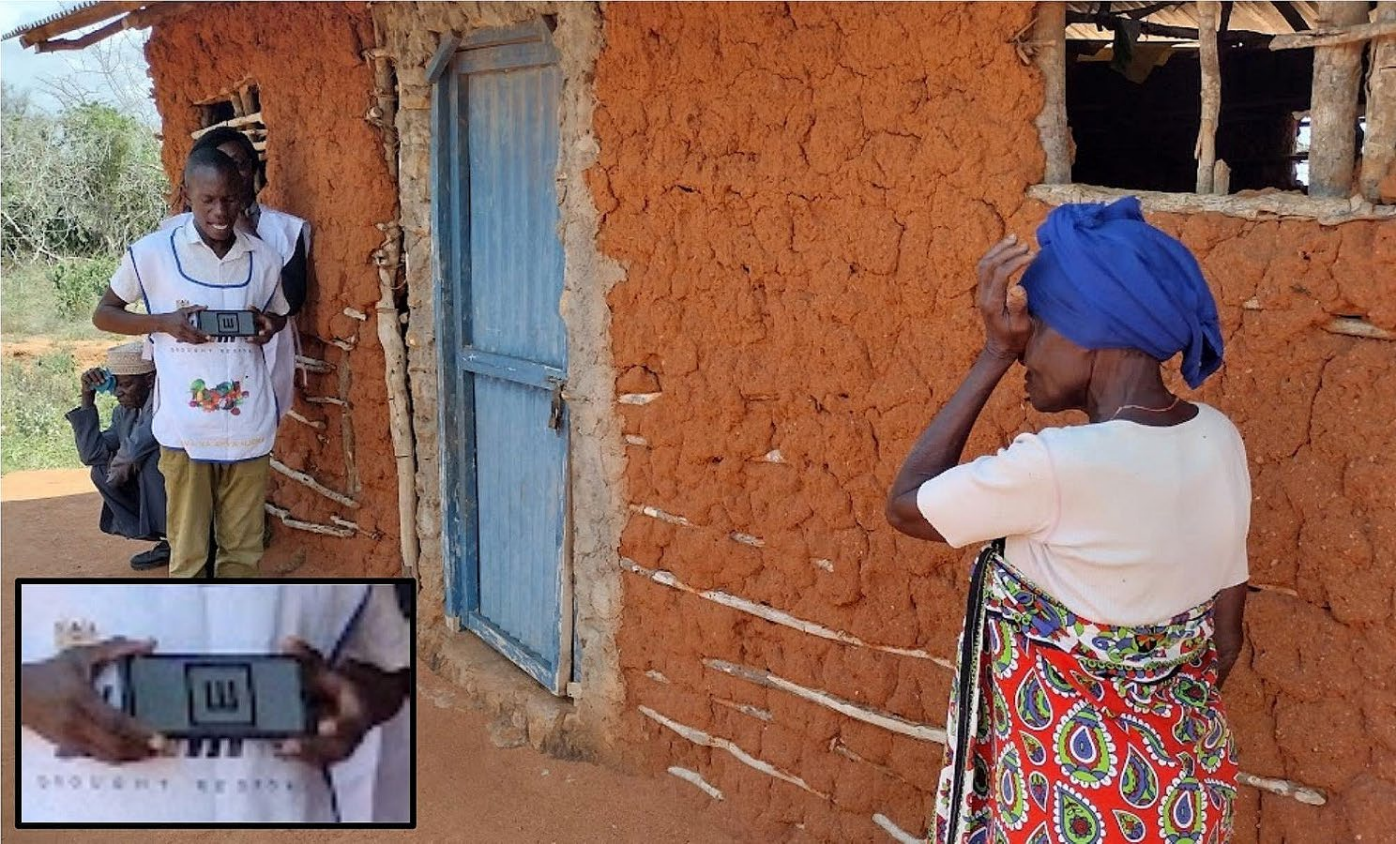
A woman having the visual acuity of her right eye screened with a ‘tumbling E’ assessment on the Peek Vision app. Caption: Eyes are tested one at a time. The screener stands 3 m away from the participant. The Peek app displays a series of letter E symbols in different sizes and orientations. The participant is asked to point in the direction that they think the E is facing (upwards in the figure). The screener swipes the screen in the direction indicated by the participant. A simple algorithm calculates visual acuity based on the number of correct swipes. Those whose vision falls below 6/12 are referred to the local outreach treatment clinic on a given date. Verbal photography consent was granted by all those in the picture

An estimated 7.5 million people require eye health services in Kenya, but less than a quarter are able to access the services they need [8]. In 2022 the government launched the ‘Vision Impact Programme’ (VIP) in which community-based teams use smartphones to administer ‘tumbling E’ visual acuity assessments, using the ‘Portable Eye Examination Kit’ (PEEK), delivered through an app that was developed by the social enterprise Peek Vision (Fig. 1) [9–12]. Those who screen positive - i.e. their visual acuity is found to fall below a predetermined threshold (< 6/12 in either eye) are referred to a local outreach treatment clinic, commonly held in a local primary care facility, where they receive free further assessment and care, including spectacles, eye drops, or onward referral for cataract surgery at a local hospital as required. Screeners also refer people who have a red eye or any other issue upon basic visual inspection, as well as anyone who feels they have an eye problem, even if there are no clinical signs.

In the VIP programme’s first year, over a million people were screened and more than 150,0000 were managed at free treatment outreach clinics [13]. Whilst this is a remarkable achievement, internal Peek data suggest that there are important issues with clinic accessibility, as less than half of those who were identified with an eye problem during community-based screening received care at their local clinic.

Access is determined by both patient and provider factors [14], and evidence from other countries suggests that certain groups such as females, widows, and those in rural areas may face unique structural barriers to accessing eye care services [15]. Currently, no sociodemographic data beyond age, gender, and language are being collected in the VIP screening programme, and these data are not currently being used to perform equity analyses. As such, any sociodemographic inequities are invisible.

Acknowledging the risk that “poorer, less advantaged segments of the population could be left behind” as countries expand access to health services in pursuit of UHC, joint WHO and World Bank guidance recommends that health programmes routinely gather data on gender, wealth, and place of residence (urban/rural) to monitor equity in effective service coverage [16]. The recent United Nations (UN) Resolution on Vision, the Lancet Commission on Global Eye Health, and the Declaration of Astana all call on global health partners to analyse the equity impact of their programs across different sociodemographic populations [1, 5, 17]. This aligns with the Sustainable Development Goal commitment ‘reach the furthest behind first’ [18].

Working with the Ministry of Health, a local community advisory board, the VIP programme implementing partner, and Peek Vision, we aimed to integrate a set of sociodemographic questions into the community-based screening process in Meru county and perform the first assessment of whether all sociodemographic groups are experiencing similar levels of access to primary eye care. These findings will inform subsequent work to address the identified inequities by understanding the barriers faced by those least likely to attend their outreach referral appointments. The findings from this study will feed into future work to understand the challenges they face, and identify and test effective service modifications to increase attendance rates among these groups, driving continuous improvement in access.

## Methods

### Population

The VIP programme has been designed to screen all residents aged over 18 years in ten of Kenya’s 47 counties [19]. Working with the national director of eye services, we selected Meru county as the most appropriate place to conduct our study, based on the fact that it contains a mix of urban and rural areas, has a leadership engaged with equity-focused quality improvement, and had a screening schedule that aligned with our research timeline. Meru is a central high-altitude county on the slopes of Mount Kenya with a population of 1.55 million, most of whom live in Meru town, the seventh largest urban centre in the country. Agriculture is the main source of employment, and khat and tea are the most prevalent cash crops. Whilst Kenya’s counties vary widely in terms of their climate, demography, and local economies, Meru’s infant mortality rate, epidemiological burden, fertility rate and education levels are closely aligned with the national average [20, 21].

### Sociodemographic domains

We started by performing a literature review and a secondary analysis of data from a systematic review to identify the sociodemographic domains that are being used by other programmes, agencies, and researchers around the world. Full details and results are available in our published protocol [22]. Briefly, we identified 11 broad domains that had been used or recommended in the peer-reviewed literature and UN agency reports: age, gender, residence (urban/rural), language, ethnicity/tribe/race/caste, refugee/immigrant status, marital status, religion, occupation, income, and wealth [1, 2, 5, 16, 23–26]. We drafted response options for each domain that aligned with those used in the widely-used United States Agency for International Development (USAID) Demographic and Health Survey (DHS) that has been used to complete more than 400 surveys in 90 countries [27, 28] and the Rapid Assessment of Avoidable Blindness (RAAB) instrument that has been used for over 300 surveys in 80 countries [29]. This was to ensure that all ensuing data complied with international norms and were maximally useful for domestic policymakers.

Next, we set up a multi-stakeholder workshop that included representatives from Peek Vision, the implementing partner organisation (Christian Blind Mission), the Ministry of Health, local academics with experience and expertise in sociodemographic data collection, and academics from the International Centre for Eye Health at the London School of Hygiene and Tropical Medicine (LSHTM). This group adapted each of the draft domains to the Kenyan context, and added a housing question as an indicator of wealth.

Over the course of four hybrid workshops, we iteratively refined the list of domains and questions stems, seeking to align them with pre-existing locally collected data and ensuring that the wording accorded with cultural norms. We removed the question on tribe/ethnicity as this was considered to be potentially inflammatory. Supplementary Tables 1–4 present further detail on the decisions made at each stage.

All decisions were made by consensus, and after five rounds of iteration the final list included 12 domains with between 2 and 8 individual response options (Table 1). Every domain also included ‘don’t know’ and ‘do not want to answer’. The draft survey instrument was translated into Kiswahili and back-translated into English to check that meaning had not been lost. The survey was piloted with laypeople using a ‘think aloud’ approach [30], and then in the actual screening programme with approximately 100 service users. No changes were indicated during piloting.

**Table 1:** Sociodemographic domains and response options

Domain	Question stem	Response options
**Gender**	What is your gender?	Female
		Male
		Other
**Age**	What is your age?	18–24
		25–34
		35–44
		45–54
		55–64
		65+
**Language**	What is your preferred language?	Kiswahili
		English
**Marital status**	What is your marital status?	Single
		Married
		Divorced/separated
		Widowed
**Assets**	Does your household own a bicycle, motorbike, scooter, car, or truck?	None
		Bike or Moto or Scooter
		Car or Tuck
**Disability**	Do you have any difficulty with hearing, walking, climbing steps or communicating?	No
		Yes (one or more)
**Education**	What is your highest level of education?	None
		Primary
		Secondary
		Post-secondary
**Health insurance**	Do you have health insurance?	No
		Yes, active
		Yes, not active
**Housing**	What is your floor made of in your house?	Cement
		Other
**Income**	In the last month, what was your approximate income?	KES < 24,000 (USD <165)
		KES 24,000–32,333 (USD 165– 220)
		KES > 32,333 (USD > 220)
**Occupation**	What is your occupation?	Not employed
		Farming
		Domestic service
		Professional*
		Sales & services
		Skilled manual
		Unskilled manual
		Student/pupil
**Religion**	What is your religion?	Christian
		Islam
		Hindu
		Other

*Note: Includes professional or manager or technician or clerical

### Screening approach

In the VIP programme, community health workers go house-to-house and assess the vision of all residents. For each participant, they enter the following demographic details into the Peek app: name, contact phone number, age, and gender. Next, they perform a ‘tumbling E’ visual acuity assessment using a smartphone. As stated above, if the participant’s vision falls below a pre-specified acuity threshold, or if they have a visible or reported subjective eye complaint (e.g. a red or painful eye), then the participant is referred to the local clinic for further assessment and treatment. At this point their preferred language is recorded. The participant is given an appointment date and is sent a follow-up reminder text message. They cannot choose the date of the appointment themselves. On the day of assessment, participants are checked-in (registered) by staff using the same Peek app at the clinic. This means that Peek hold a record of all those referred and can generate a complete list of all those who have and have not been checked-in on their appointed date. Meru county has 11 sub-counties. The VIP programme leaders organise screening and treatment at the sub-county level. This study reports on findings from the Tigania West sub-county, a peri-urban region with community units and villages distributed throughout. All villages in the area were visited for screening and 50 treatment outreach centres (clinics) were identified and used as triage centres. Each community unit has approximately 4,000 people residing in it, generating approximately 300–400 referrals over the analysis period. Triage centres are generally held in primary care facilities, churches, or other meeting spaces. They are run by a small team of optometrists and allied eye health professionals.

Participants were distributed across the entire sub-county, and screening of those included in this analysis (*n* = 4, 240) was conducted between April and July 2023. Referred patients generally queue to be seen, and hundreds of people can be treated each day. Refraction services, eye drops, and other medications can be administered, along with further referrals for specialist eye care when needed. All care is free.

We added the extended list of sociodemographic questions to the Peek app. These questions were asked of every consenting person who was found to have an eye problem and referred to their local treatment outreach clinic. Informed written consent to gather these additional sociodemographic data was obtained by the community health workers who performed the screening.

### Sample size

Our aim was to compare the odds of attendance between different sociodemographic subgroups (e.g. males vs. females). Our community advisory group suggested that we should aim to detect differences in attendance of 5–10% or more between subgroups. With a 95% confidence level and a maximally conservative proportion of 50% attendance, we calculated that we would need to have at least 1,566 people in each subgroup to have 80% power to detect a 5% difference between subgroups, or 385 people in each subgroup to detect a difference of 10%. We decided to set our sample size at 3,850 which would provide 80% power to detect differences of 10% between groups that contain at least 10% of the overall population, while still providing power to detect a difference of 5% in subgroups that make up 40% of the population. We deemed that this would enable robust comparisons between most subgroups, and accepted that we would only be able to identify large differences between subgroups that contained very few people e.g. those in the highest income category or those reporting a religion other than Christianity or Islam.

We reviewed the number of people who had been recruited on a weekly basis and stopped data collection on the day that the sample exceeded 3,850.

### Outcome

Our primary outcome was clinic attendance, which we used as a proxy for access. People who are referred to clinics are given an appointment data that is generally 1–2 weeks after the date of screening, however people occasionally present up to a week after their appointed date. As such, we decided to use attendance at triage clinic within 21 days (including weekends) of referral as our primary outcome. Participants who entered the screening programme within three weeks of the end of the programme were excluded from the analysis.

### Statistical analysis

We used logistic regression to calculate the adjusted odds of non-attendance for each sociodemographic subgroup. Our statistical approach is outlined below:

1. Perform simple logistic regression with attendance as the outcome. Separately add each sociodemographic domain as an exposure. (Unadjusted model)
2. Adjust each model for age and gender. (Minimally adjusted model)
3. Adjust each model for all other sociodemographic variables. (Fully adjusted model)
4. Test an interaction between each sociodemographic variable and age category (Effect modification by age).
5. Test an interaction between each sociodemographic variable and gender (Effect modification by gender).

### Post-hoc sensitivity analyses

To quantify the impact of intersectionality [31, 32], we estimated the probability of attendance for people with different combinations of sociodemographic characteristics that were found to be the strongest predictors for poor access.

After completing our analysis, our Kenyan Ministry of Health collaborators sensibly hypothesized that severity of eye condition could explain differences in attendance by age and other sociodemographic domains, reasoning that those with painful or severe conditions might be more likely to seek care than those with mild or painless conditions. Data on eye conditions had already been collected during screening. We categorised these diagnostic codes into five categories that grouped conditions based on their likely acuity and impact (below). Then we re-ran the regression models to control adjust for these additional eye condition data.

- Normal vision.
- Loss of vision (visual acuity < 6/12 vision in either eye).
- Chronic problem: Growth on eyeball, Lump on lids, White pupil, Strabismus.
- Acute problem: Conjunctivitis, Redness, Redness with discharge, Red and watery itchy eye.
- Urgent problem: Eye injury, Pain, Marked swelling of the eyelids.

### Bias

To reduce the risk of selection bias, the sociodemographic questions were asked of every consecutive person who was referred until we had collected data from at least 3,850 people. We developed a robust set of questions to minimise the risk of recall bias, grounded in the literature and tailored to the local context by a group of experts and community representatives. We delivered standardised training to the data collectors in order to minimise the risk of measurement bias. We also performed unannounced observations of screeners to check that the questions were being asked as intended. We found no fidelity issues during these visits.

## Results

Between April and July 2023, 136,912 people aged > 18 years old were screened in Meru County and 32,835 people were found to have an eye problem that required referral to a local treatment outreach clinic (24.0% of all those screened). We gathered and analysed data from the first 4,240 referred people who consented to provide their sociodemographic information. As several hundred people were screened every week, our final sample exceeded 3,850.

We found that just under half were able to access their appointment (46.0%). In our fully adjusted model, we found very strong evidence (*p* < 0.001) of an association between three variables and access: gender, with males found to be less likely to access care than females; age, with younger adults less likely to access care than older adults; and occupation, where those in skilled/unskilled manual jobs and sales & services had the lowest access. Younger adults had the worst access overall, with only 32% of those aged 18–44 years being checked-in at clinics compared to 54% of those aged ≥ 45 years old.

Three other variables showed some weaker evidence of an association with the outcome; education (*p* = 0.03), marital status (*p* = 0.03), and vehicle ownership (*p* = 0.03) (Table 2).

**Table 2:** Attendance by sociodemographic group

		*N*	*N* Attended	% Attended	Unadjusted OR	*p*-value	Adjusted for age and gender	*p*-value	Adjusted for everything	*p*-value
**Gender**	Female	2700	1317	49%	Ref	< 0.001	Ref	< 0.001	Ref	< 0.001
	Male	1540	634	41%	0.73 (0.65–0.83)		0.67 (0.59–0.76)		0.72 (0.63–0.83)	
**Age**	18–24	271	78	29%	0.42 (0.32–0.57)	< 0.001	0.41 (0.31–0.55)	< 0.001	0.49 (0.35–0.69)	< 0.001
	25–34	615	189	31%	0.46 (0.38–0.57)		0.45 (0.36–0.55)		0.51 (0.41–0.63)	
	35–44	730	256	35%	0.57 (0.47–0.69)		0.55 (0.46–0.67)		0.59 (0.48–0.72)	
	45–54	1048	512	49%	Ref		Ref		Ref	
	55–64	786	429	55%	1.26 (1.05–1.51)		1.27 (1.05–1.53)		1.21 (1.00-1.46)	
	65+	790	487	62%	1.68 (1.39–2.03)		1.71 (1.42–2.07)		1.61 (1.31–1.99)	
**Transport assets**	None	3644	1726	47%	Ref	0.0001	Ref	0.002	Ref	0.03
	Bike/Moto/scooter	328	125	38%	0.68 (0.54–0.86)		0.86 (0.68–1.10)		0.87 (0.68–1.12)	
	Car	268	100	37%	0.66 (0.51–0.85)		0.64 (0.49–0.83)		0.69 (0.52–0.92)	
**Disability**	No	3637	1629	45%	Ref	< 0.001	Ref	0.87	Ref	0.99
	Yes	603	322	53%	1.41 (1.19–1.68)		0.98 (0.82–1.18)		1.00 (0.83–1.20)	
**Education**	None	284	149	52%	Ref	< 0.001	Ref	0.002	Ref	0.03
	Primary	1787	906	51%	0.93 (0.73–1.20)		1.43 (1.09–1.87)		1.42 (1.07–1.87)	
	Secondary	1538	666	43%	0.69 (0.54–0.89)		1.28 (0.97–1.69)		1.30 (0.97–1.73)	
	Post-secondary	631	230	36%	0.52 (0.39–0.69)		1.03 (0.76–1.40)		1.12 (0.81–1.56)	
**Health insurance**	No	2530	1154	46%	Ref	0.35	Ref	0.77	Ref	0.12
	Yes, active	909	437	48%	1.10 (0.95–1.28)		1.02 (0.87–1.19)		1.20 (1.01–1.43)	
	Yes, not active	801	360	45%	0.97 (0.83–1.14)		0.95 (0.80–1.12)		1.04 (0.88–1.24)	
**Cement floor**	No	703	353	50%	Ref	0.015	Ref	0.21	Ref	0.48
	Yes	3537	1598	45%	0.82 (0.69–0.96)		0.90 (0.76–1.06)		0.94 (0.79–1.12)	
**Monthly income (KES)**	No response	1984	935	47%	Ref	< 0.001	Ref	0.007	Ref	0.11
	< 24,000	2050	939	46%	0.94 (0.84–1.07)		0.92 (0.81–1.04)		0.91 (0.80–1.04)	
	24,000–32,333	132	56	42%	0.83 (0.58–1.18)		0.84 (0.59–1.22)		0.98 (0.67–1.45)	
	> 32,333	74	21	28%	0.44 (0.27–0.74)		0.41 (0.24–0.69)		0.54 (0.30–0.95)	
**Marital status**	Single	904	320	35%	Ref	< 0.001	Ref	0.005	Ref	0.03
	Married	2977	1435	48%	1.96 (1.64–2.33)		1.37 (1.12–1.66)		1.29 (1.05–1.59)	
	Divorced/separated	200	93	47%	1.83 (1.33–2.51)		1.12 (0.79–1.57)		1.10 (0.77–1.55)	
	Widowed	333	185	56%	2.63 (2.01–3.41)		1.05 (0.77–1.42)		1.03 (0.76–1.42)	
	Other	26	11	42%	1.54 (0.70–3.41)		0.87 (0.38–1.97)		0.89 (0.38-2.00)	
**Occupation**	Not employed	801	367	46%	Ref	< 0.001	Ref	< 0.001	Ref	< 0.001
	Farming	1593	892	56%	1.50 (1.27–1.78)		1.29 (1.08–1.54)		1.24 (1.03–1.49)	
	Domestic service	297	162	55%	1.42 (1.09–1.85)		1.45 (1.10–1.91)		1.44 (1.09–1.90)	
	Professional	202	79	39%	0.76 (0.55–1.04)		0.86 (0.62–1.19)		1.05 (0.73–1.52)	
	Sales & services	449	151	34%	0.60 (0.47–0.76)		0.73 (0.56–0.93)		0.76 (0.58–0.98)	
	Skilled manual	400	138	35%	0.62 (0.49–0.80)		0.78 (0.60–1.01)		0.79 (0.60–1.04)	
	Unskilled manual	417	140	34%	0.60 (0.47–0.76)		0.72 (0.56–0.93)		0.72 (0.55–0.93)	
	Student/pupil	81	22	27%	0.44 (0.27–0.73)		0.86 (0.49–1.51)		1.00 (0.56–1.77)	
**Religion**	Christian	4129	1907	46%	Ref	0.09	Ref	0.15	Ref	0.24
	Islam	81	36	44%	0.93 (0.60–1.45)		0.95 (0.60–1.50)		1.07 (0.67–1.69)	
	Other	30	8	27%	0.42 (0.19–0.95)		0.44 (0.19-1.00)		0.49 (0.21–1.14)	

**Fig. 2:**
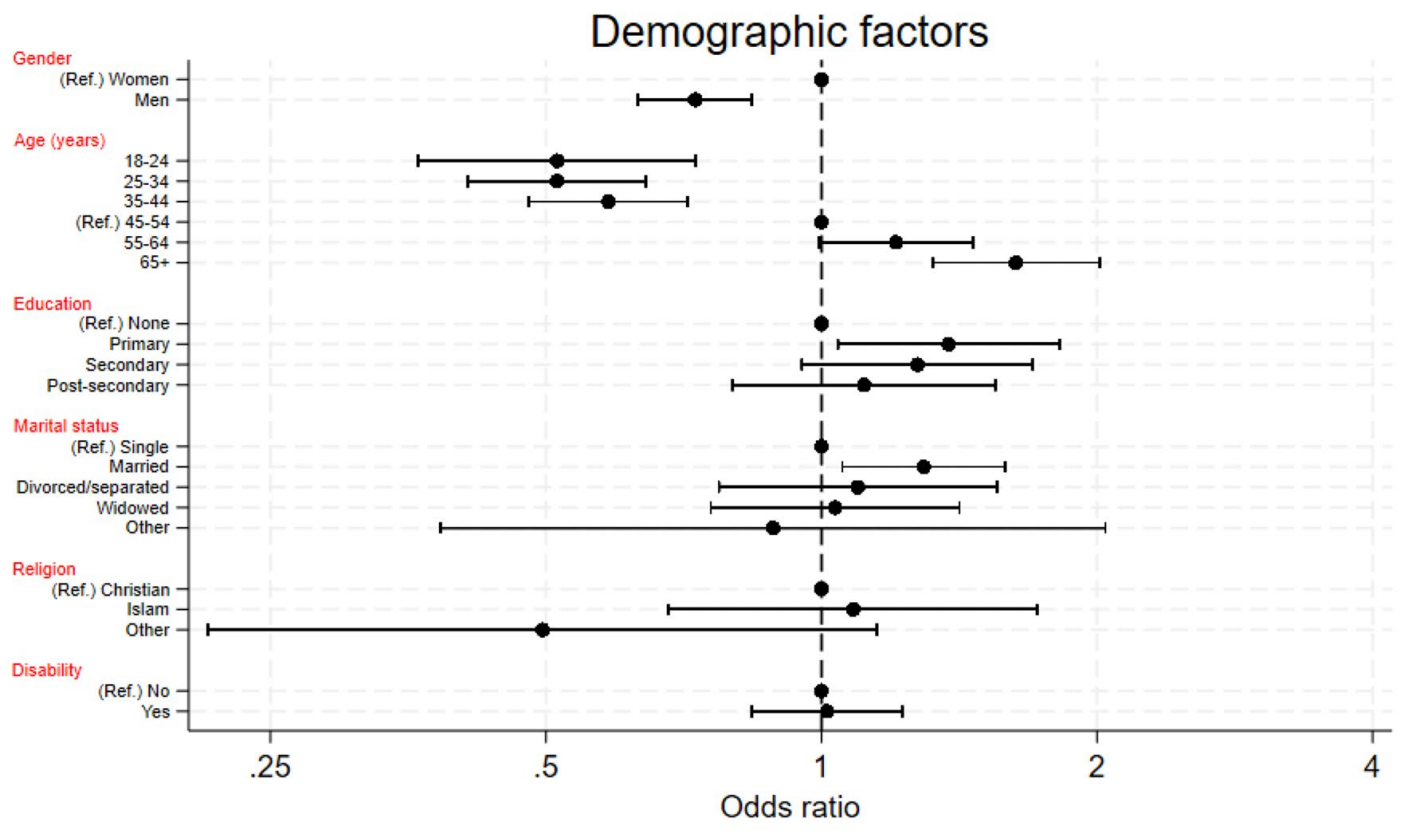
Plot of fully adjusted odds ratios of attendance according to demographic factors. Ref. = Reference group, disability = yes means the participant responded that they had difficulty with at least one of hearing, walking, climbing steps or communicating

**Fig. 3:**
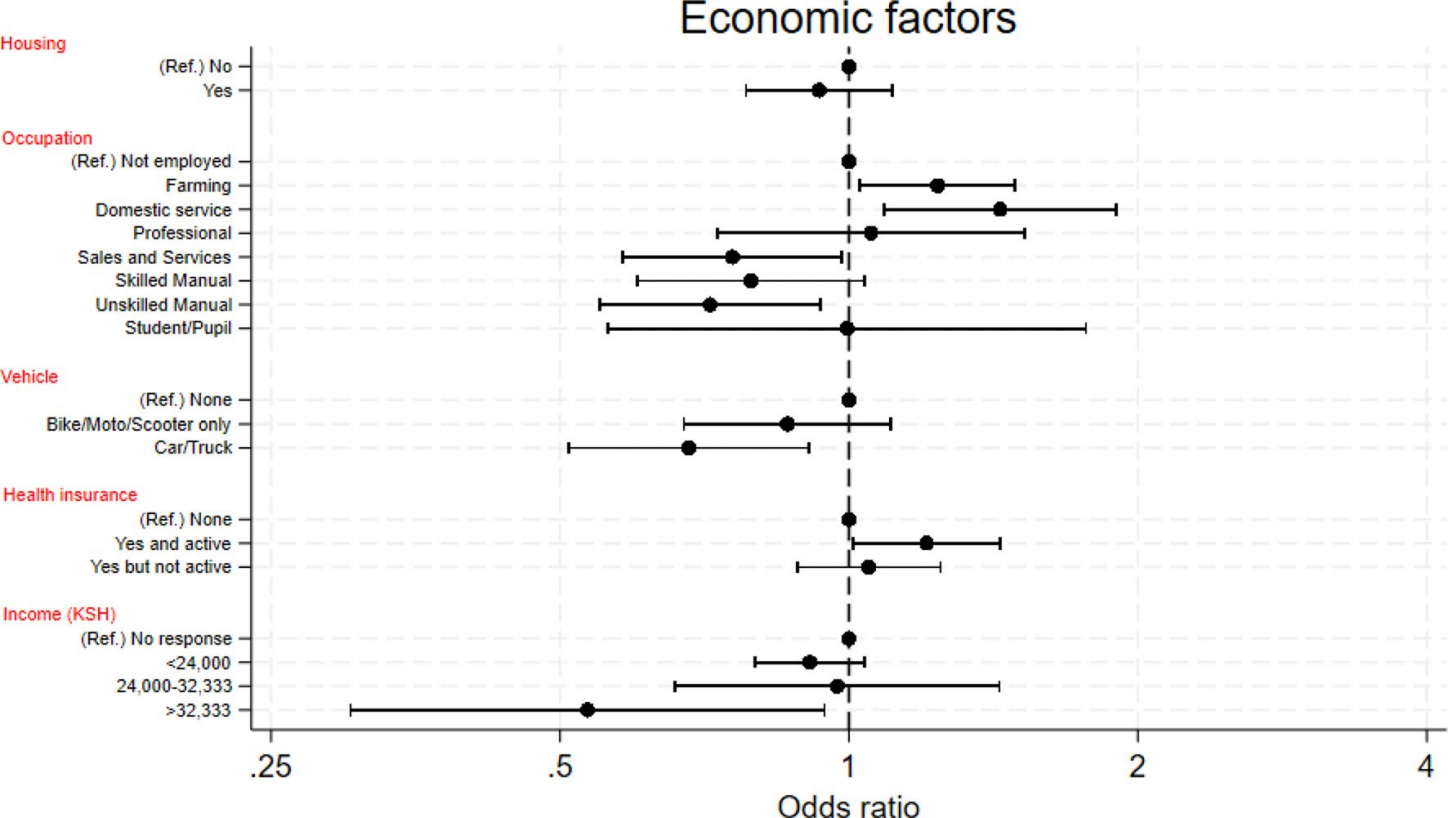
Plot of fully adjusted odds ratios of attendance according to economic factors. Ref. = Reference group

Figures 2 and 3 plot the adjusted odds ratios of attendance for the demographic and economic factors.

**Fig. 4:**
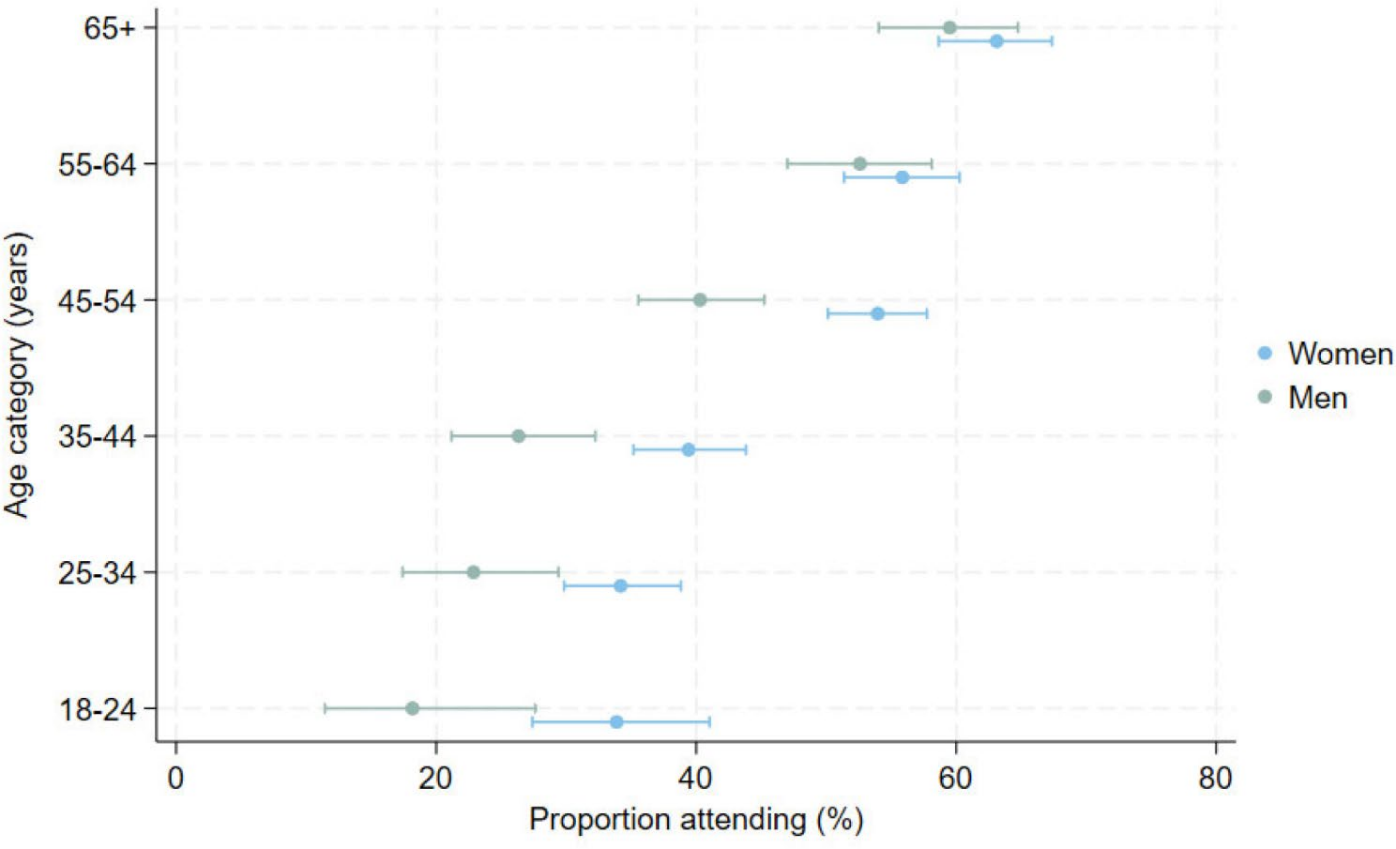
Clinic attendance within each age and gender group

We tested for effect modification and identified some weak evidence (*p* = 0.05) of an interaction between age and gender, suggesting that the difference in attendance between men and women is greater at younger ages than in older (Fig. 4 and Supplementary Table 5).

### Sensitivity analyses

To quantify the impact of intersectionality, we estimated the probability of attendance for people with different combinations of age, gender (including the interaction between age and gender), and occupation – the three strongest predictors of access. Age and gender were already categorical variables. For simplicity, we dichotomised occupation into a binary variable, grouping together the three categories of occupation that had the lowest attendance (skilled/unskilled manual and sales & services).

We found that the expected lowest attending group is 18-24-year-old males who work in sales/service/manual jobs, where we estimate that only 14% of people with these three characteristics would be able to access care (95% CI: 8 to 22%). However, there were only 29 people with all three of these characteristics i.e. this group constituted < 1% of all those referred. The highest estimated access rate was 64%, found among females aged 65 + not working in those occupations (95% CI: 59–68%). This group represented 11% of all those referred.

In our second sensitivity analysis we adjusted for severity of eye condition. We found that eye condition did not affect the effect estimates, suggesting that this variable was not driving greater attendance in older people (Supplementary Table 6).

## Discussion

The growing emphasis on extending Universal Health Coverage and ‘leaving no-one behind’ means that programme managers around the world are increasingly being expected to identify populations that face unique barriers to care. Aligning with findings from previous research in Kenya [10], we found that less than half of all people who screened positive in Meru’s VIP project were able to access care. This resonates with a 2018 systematic review that found that 43% of all African outpatient appointments are missed, with younger adults and those from lower socioeconomic groups being the least likely to access care [33].

We found that younger men working in sales, services, or manual jobs were the least likely to be checked-in at treatment clinics in Meru. This was similar to findings reported in Mozambique where manual and domestic workers were less likely to have their eye examinations up to date [34]. In contrast, findings from other settings on access to eye services which has shown older age, female gender, and widowhood to be the strongest predictors of poor access [1, 15]. However, these previous studies conducted in Nigeria and Sri Lanka have focused on cataract care which affects people later on in life, whereas the VIP programme addresses all eye conditions in all ages. We note that our study adjusted for age and eye condition.

Given that Kenya ranks 110th out of 144 countries in the UN’s gender equality ranking [35], we were surprised that men were 30% less likely to attend than women in the fully adjusted model. However, this is not an unusual finding. Despite having greater power, privileges, and opportunities than women in virtually all societies, men commonly experience higher rates of poor health, lower rates of health care access, and lower overall life expectancy [36, 37]. Differences in healthcare-seeking behaviour are thought to drive much of the gender gap in access to care, related to differences in perception of risk and pervasive social ideals of masculinity [38]. We do not have any data to suggests that these themes underly the gender difference observed in Meru.

Whilst younger men were the least likely to reach the clinics in Meru, younger women were less likely to attend than older women, suggesting that youth is an important independent factor. In fact, age was by far the strongest predictor overall, with the youngest cohort (18-24y) three times less likely to have been checked-in than the oldest (65+), even after adjusting for gender, occupation, and severity of eye condition.

We hypothesise that younger adults may be more likely to be ‘hustling’ than older people – i.e. working in informal jobs with no fixed salary or paid sick leave, and therefore facing higher financial opportunity costs when taking time out to attend a clinic. The fact that people working in (often informal) sales, services, and manual labour were also less likely to attend than those working in other areas seems to corroborate this hypothesis.

To a lesser extent, car/truck ownership and high level of income were also associated with poor access. We hypothesise that this may be because more affluent people who are told they have an eye problem at screening may be seeking private care rather than attending the free public VIP clinics. However, Kenya’s top income tax rate (used to delineate high income earners) is set at the equivalent of USD 2,660/year which only slightly above the national median income. As such it is unclear what proportion of the ‘high earners’ who own cars and trucks are actually meaningfully better-off than those in the middle-income group. We plan to conduct a set of interviews with people from this group to explore this issue further. Future iterations of this analysis will use a higher income threshold designed to capture those at the top end of the income distribution.

Our study had a number of other limitations. We did not include questions on religion, tribe/ethnicity, or sexuality due to concerns about cultural sensitivities, but these are all important markers of potential access challenges [24, 25]. With a larger sample we would have been able to detect smaller differences between groups, however it would have taken longer to conduct the study and the embedded nature of this research comes with pressure to deliver rapid and timely findings. We focused on contact coverage (access to clinics) rather than effective coverage (receipt of services of sufficient quality to obtain potential health gains [39]) which is arguably a more important metric. [40, 41]. This decision was driven by feasibility, given that check-in data are routinely collected but clinical outcome data are not. However, this is an important avenue for future work. The eye condition categories are entered by screeners who do not have specialist ophthalmic training, and therefore may not code all patients appropriately. Furthermore, eye conditions affect younger and older people in different ways. Future qualitative work will help to uncover the specific barriers that prevent younger people from accessing services in Meru. Finally, we have not yet validated our sociodemographic questions. This work is currently underway, however the process of selecting the items and response options was based on extensive literature review and wide stakeholder engagement to ensure that we were using previously-validated questions with strong external validity.

## Conclusions

Less than half of those referred to local eye clinics were able to access care. We found evidence of large sociodemographic inequalities, with younger adults, males, and those working in sales, services, and manual jobs the least likely to access care. Overall, age was the strongest predictor. Future work should focus on exploring the specific barriers faced by younger adults and their ideas for how services could be modified to improve access to community-based eye services.

### Electronic supplementary material

Below is the link to the electronic supplementary material.

Supplementary Material 1

## Data Availability

The datasets used and/or analysed during the current study are available from the corresponding author on reasonable request. Individual-level data will be pseudo-anonymised, removing names and any other key identifiers before it is shared. Only the least amount of data will be shared, and where possible it will be fully anonymised and aggregated. All published findings will be at anonymous aggregate subpopulation level.

## Abbreviations

CI: Confidence Interval
DHS: Demographic and Health Survey
KEMRI: Kenya Medical Research Institute
LSHTM: London School of Hygiene and Tropical Medicine
ODA: Official Development Assistance
OR: Odds Ratio
RAAB: Rapid Assessment of Avoidable Blindness
UHC: Universal Health Coverage
UN: United Nations
USAID: United States Agency for International Development
VIP: Vision Impact Programme
WHO: World Health Organization
