# Supplementary material for: Access to community-based eye services in Meru, Kenya: a cross-sectional equity analysis
Source: Int J Equity Health. 2024 Aug 26;23:170. doi: 10.1186/s12939-024-02244-x (PMC11346173; doi:10.1186/s12939-024-02244-x)

# Appendix

## Supplementary Tables 1-4: Sociodemographic variable section process

**Supplementary Table 1: Sociodemographic variables from the first multi-stakeholder workshop**

| **Domain**  **(Data type)** | **Adult response options** | **Notes** |
| --- | --- | --- |
| Age (years)  (Discrete) | Any integer >18 | Already routinely collected in all Peek programmes |
| Gender  (Categorical) | - Female - Male - Other | Already routinely collected in all Peek programmes  The DHS and RAAB7 surveys only include female/male. We have added ‘other’ |
| Phone ownership  (Ordinal) | Do you need someone else to receive your text message reminders?   - Yes, my mother or father - Yes, my spouse - Yes, my daughter or son - Yes, other - No (= phone ownership) | Already routinely collected in all Peek programmes |
| Place of residence  (Categorical) | N/A | Urban/rural location automatically inferred from screening location |
| Distance from screening location to clinic (km)  (Discrete) | N/A | Distance between screening location and clinic location has been found to be a predictor of outcomes  This is automatically calculated by the Peek software. |
| Language  (Categorical) | - [list languages] | Country-specific lists will be derived from the latest Demographic and Health Survey |
| Relationships  (Categorical) | - Married or living together - Divorced/separated - Widowed - Never married or lived together | Options may need tailoring depending on the context. |
| Ethnicity  (Categorical) | - [List ethnic groups] - Other | Country-specific lists will be derived from the latest Demographic and Health Survey |
| Migrant/refugee  (binary) | Are you a migrant or refugee?   - Yes - No | May be inflammatory depending on the setting |
| Religion  (Categorical) | - [List main religions] - Other not listed - None | Country-specific lists will be derived from the latest Demographic and Health Survey |
| Education  (Ordinal) | - None/pre-school only - Non-formal (included Quranic) - Some primary - Completed primary - Some secondary - Completed secondary - University | Options taken from the RAAB7 survey as it offers more detail than the DHS model questionnaire (early childhood education programme/Primary/Secondary/Higher)  Non-formal/Quranic options may not be appropriate in settings where the prevalence of these forms is negligible |
| Occupation  (Ordinal) | - Unemployed - Unskilled manual - Skilled manual - Professional - Homemaker | For children, programme implementers will ask what their parent’s do for work and then code the highest occupational category on their behalf |
| Income (proxy)  (Ordinal) | When you think about the food in your household would you say you have:   - Less than adequate food for the needs of the household - Just adequate - More than adequate | This question is being used in the RAAB7 eye health survey as a proxy for income  The survey is designed for >50y olds, so the response options may not be appropriate for children |
| Income adequacy  (Ordinal) | When you think about the income in your household would you say it is:   - Not enough to cover our needs, we must borrow, - Not enough to cover our needs, we use savings, - Just enough to cover our needs, - Enough to cover our needs, we are able to save a little - Enough to cover our needs, we are building savings | This question is being used in the RAAB7 eye health survey as a proxy for income  The survey is designed for >50y olds, so the response options may not be appropriate for children |
| Wealth  (Binary) | Is your house’s floor made out of cement?   - Yes - No | The specific indicator used here will depend on the location |
| Assets  (Binary) | Does your household own:   - [List assets from DHS] | Shortest possible list of assets to be selected by country working groups |

**Note**: Every question will have the additional options: ‘Do not want to answer’ and ‘Don’t know’.

**Supplementary Table 2: Sociodemographic variables from the second multi-stakeholder workshop**

| **Domain** | **Adult response options** | **Notes** |
| --- | --- | --- |
| Age | Any integer >18 | Already routinely gathered |
| Gender | •Female  •Male  •Other | Already routinely gathered |
| Phone  ownership | Do you need someone else to receive your text?  message reminders?   - Mother or father - Spouse - Daughter or son - Other - No (=phone ownership) | Already routinely gathered |
| Place of  residence | N/A | Urban/rural automatically  inferred |
| Distance to  clinic | N/A | Automatically calculated by  Peek |
| Language | What language do you speak most often at home?  •English  •Swahili  •Borana  •Embu  •Kalenjin  •Kamba  •Kikuyu  •Kisii  •Luhya  •Maragoli  •Luo  •Maasai  •Meru  •Mijikenda  •Pokot  •Somali  •Turkana  •Other | Workshop participants felt  that it would be inflammatory  to ask about tribe/ethnicity.  Language will be used as a  Proxy |
| Relationships | •Never married  •Married  •Living together  •Single  •Divorced/separated  •Widowed |  |
| Migrant status | Were you born in Kenya?  •Yes  •No  •Don’t want to answer | This question may be  redundant. Kenya is currently  home to 500,000 refugees,  however, they mainly live in  camps and this information  will already be collected under  ‘Place of residence’. Outside  of Nairobi, the migrant  population that does not live  in camps is negligible. |
| Religion | What is your religion?  •Roman Catholic  •Protestant/other Christian  •Islam  •Other  •No religion | Responses taken from the  2014 DHS |
| Education | What is you highest level of completed schooling?  •No education  •Some primary  •Primary complete  •Some secondary  •Secondary complete  •More than secondary | Adult responses aligned with the 2014 DHS |
| Occupation | What is your occupation?  •Unemployed  •Agriculture  •Unskilled manual  •Skilled manual  •Sales and services  •Clerical  •Professional/technical/managerial  •Homemaker | Interviewer to categorise and  code the highest |
| Food adequacy | When you think about the food in your  household would you say you have:  • Less than adequate food for the needs of the household  •Just adequate  •More than adequate | Question taken from RAAB7  – may remove due to poor face validity |
| Income  adequacy | When you think about the income in your  household would you say it is:  • Not enough to cover our needs, we must borrow,  • Not enough to cover our needs, we use savings,  • Just enough to cover our needs,  • Enough to cover our needs, we are able to save a little  • Enough to cover our needs, we are building savings | From RAAB7, but poor face  validity. |
| Housing | Is your house’s floor made of earth, sand, or dung?  •Yes  •No  Do you have water piped into your own house or yard?  •Yes  •No  Does your household have electricity?  •Yes  •No  What kind of toilet does your household you use?  •Own toilet/latrine  •Shared toilet/latrine  •None (bush/field) | All options taken from the  2014 DHS |
| Assets | Do you own a smartphone?  •Yes  •No  Does your household own a:  •Bicycle  •Motorcycle/scooter  •Car or truck  Do you own your dwelling?  •Yes  •No |  |

**Supplementary Table 3: Sociodemographic variables from the third multi-stakeholder workshop**

| **Domain** | **Adult response options** | **Child response options** | **Notes** |
| --- | --- | --- | --- |
| Age | Any integer >18 | Any integer 5 - 17 | Already routinely gathered |
| Gender | •Female  •Male  •Other | •Female  •Male  •Other | Already routinely gathered |
| Phone  ownership | Do you need someone else to receive your text?  message reminders?   - Mother or father - Spouse - Daughter or son - Other - No (=phone ownership) | Provided contact number:   - Mother or father - Guardian - Teacher - Other | Already routinely gathered |
| Place of  residence | N/A | N/A | Urban/rural automatically  inferred |
| Distance to  clinic | N/A | N/A | Automatically calculated by  Peek |
| Language | What language do you speak most often at home?  •English  •Swahili  •Borana  •Embu  •Kalenjin  •Kamba  •Kikuyu  •Kisii  •Luhya  •Maragoli  •Luo  •Maasai  •Meru  •Mijikenda  •Pokot  •Somali  •Turkana  •Other | What language do you speak most often at home?  •English  •Swahili  •Borana  •Embu  •Kalenjin  •Kamba  •Kikuyu  •Kisii  •Luhya  •Maragoli  •Luo  •Maasai  •Meru  •Mijikenda  •Pokot  •Somali  •Turkana  •Other | Used instead of ethnicity |
| Relationships | •Never married  •Married  •Living together  •Single  •Divorced/separated  •Widowed | *Do you live with:*   - Both parents - Just one parent - Another relative - Guardian (non-relative) - Orphanage |  |
| Religion | What is your religion?  •Roman Catholic  •Protestant/other Christian  •Islam  •Other  •No religion | What is your religion?  •Roman Catholic  •Protestant/other Christian  •Islam  •Other  •No religion | Responses taken from the  2014 DHS |
| Education | What is you highest level of completed schooling?  •No education  •Some primary  •Primary complete  •Some secondary  •Secondary complete  •More than secondary | N/A | Adult responses aligned with the 2014 DHS |
| Occupation | What is your occupation?  •Unemployed  •Agriculture  •Unskilled manual  •Skilled manual  •Sales and services  •Clerical  •Professional/technical/managerial  •Homemaker | What are your parents’ jobs?  •No parents  •Unemployed  •Agriculture  •Unskilled manual  •Skilled manual  •Sales and services  •Clerical  •Professional/technical/managerial  •Homemaker | Interviewer to categorise and  code the highest |
| Income  adequacy | When you think about the income in your  household would you say it is:  • Not enough to cover our needs, we must borrow,  • Not enough to cover our needs, we use savings,  • Just enough to cover our needs,  • Enough to cover our needs, we are able to save a little  • Enough to cover our needs, we are building savings | N/A | From RAAB7, but poor face  validity. Agree to drop and replace with actual monthly income thresholds. |
| Housing | Is your house’s floor made of earth, sand, or dung?  •Yes  •No  Do you have water piped into your own house or yard?  •Yes  •No  Does your household have electricity?  •Yes  •No  What kind of toilet does your household you use?  •Own toilet/latrine  •Shared toilet/latrine  •None (bush/field) | Is your house’s floor made of earth, sand, or dung?  •Yes  •No  Do you have water piped into your own house or yard?  •Yes  •No  Does your household have electricity?  •Yes  •No  What kind of toilet does your household you use?  •Own toilet/latrine  •Shared toilet/latrine  •None (bush/field) | All options taken from the  2014 DHS |
| Assets | Do you own a smartphone?  •Yes  •No  Does your household own a:  •Bicycle  •Motorcycle/scooter  •Car or truck  Do you own your dwelling?  •Yes  •No | Does your household own a  smartphone?  •Yes  •No  Does your household own a:  •Bicycle  •Motorcycle/scooter  •Car or truck |  |

**Supplementary Table 4: Sociodemographic variables from the fourth multi-stakeholder workshop**

| **Domain** | **Adult response options (>18y)** | **Child response options** | **Notes** |
| --- | --- | --- | --- |
| Age | How old are you? | How old are you | Already routinely gathered |
| Gender | •Female  •Male  •Other | •Female  •Male  •Other | Already routinely gathered |
| Phone  ownership | Do you need someone else to receive your text message reminders?  •Mother or father  •Spouse  •Daughter or son  •Other  • No (= phone ownership) | Provided contact number:  •Mother or father  •Guardian  •Teacher  •Other | Already routinely gathered |
| Place of  residence | N/A | N/A | Urban/rural automatically inferred |
| Distance to  clinic | N/A | N/A | Automatically calculated by Peek |
| Language | What is your mother tongue?  •English  •Swahili  •Borana  •Embu  •Kalenjin  •Kamba  •Kikuyu  •Kisii  •Luhya  •Maragoli  •Luo  •Maasai  •Meru  •Mijikenda  •Pokot  •Somali  •Turkana  •Other | What is your mother tongue?  •English  •Swahili  •Borana  •Embu  •Kalenjin  •Kamba  •Kikuyu  •Kisii  •Luhya  •Maragoli  •Luo  •Maasai  •Meru  •Mijikenda  •Pokot  •Somali  •Turkana  •Other |  |
| Relationships | •Married  •Single  •Divorced/separated  •Widowed  •Other | *Do you live with:*  •Both parents  •Just one parent  •Another relative  •Guardian (non-relative)  •Orphanage | We removed ‘never married’  because this is the same as single  We removed ‘living together’  because this question is loaded  with social stigma  Ideally, we would ask children if  one or more parent had died,  but we don’t want to cause  distress. In the future we could  consider asking teachers for this  information |
| Religion | What is your religion?  •Christian  •Islam  •Hindu  •Other | What is your religion?  •Christian  •Islam  •Hindu  •Other | We removed ‘no religion’ as this  group is negligible  Christian denominations were  aggregated, and we added ‘Hindu’ |
| Education | What is you highest completed level of schooling?  •No education  •Primary  •Secondary  •Post-secondary | N/A | We reworded the question and  removed ‘completed’ and ‘some’  options to simplify the list |
| Disability | Do you have difficulty **hearing**, even if using a hearing aid(s)?  • No difficulty  • Some difficulty  • A lot of difficulty  • Cannot do at all  • Don’t know  Do you have difficulty **walking or climbing steps**?  • No difficulty  • Some difficulty  •A lot of difficulty  •Cannot do at all  •Don’t know  Do you have difficulty **remembering or concentrating?**  • No difficulty  • Some difficulty  •A lot of difficulty  •Cannot do at all  •Don’t know  Do you have difficulty with **self-care**, such as washing all over or dressing?  • No difficulty  • Some difficulty  •A lot of difficulty  •Cannot do at all  •Don’t know  Using your language, do you have difficulty **communicating,** for example understanding or being understood?  • No difficulty  • Some difficulty  •A lot of difficulty  •Cannot do at all  •Don’t know | Do you have difficulty **hearing**, even if using a hearing aid(s)?  • No difficulty  • Some difficulty  • A lot of difficulty  • Cannot do at all  • Don’t know  Do you have difficulty **walking or climbing steps**?  • No difficulty  • Some difficulty  •A lot of difficulty  •Cannot do at all  •Don’t know  Do you have difficulty **remembering or concentrating?**  • No difficulty  • Some difficulty  •A lot of difficulty  •Cannot do at all  •Don’t know  Do you have difficulty with **self-care**, such as washing all over or dressing?  • No difficulty  • Some difficulty  •A lot of difficulty  •Cannot do at all  •Don’t know  Using your language, do you have difficulty **communicating,** for example understanding or being understood?  • No difficulty  • Some difficulty  •A lot of difficulty  •Cannot do at all  •Don’t know | New question added at the  request of implementing partners  Response options taken from the  Washington Group Short Set on  Functioning:  [https://www.washingtongroup-](https://www.washingtongroup-disability.com/question-sets/wg-short-set-on-functioning-wg-ss/)  [disability.com/question-sets/wg-](https://www.washingtongroup-disability.com/question-sets/wg-short-set-on-functioning-wg-ss/)  [short-set-on-functioning-wg-ss/](https://www.washingtongroup-disability.com/question-sets/wg-short-set-on-functioning-wg-ss/)  The same options will be used for  adults and children. UNICEF does  have a child-specific question set,  but it is more than double the  length. |
| Occupation | What is your occupation?  •Not employed  •Agriculture  •Domestic service  •Unskilled manual  •Skilled manual  •Sales and services  •Clerical  •Professional/technical/managerial | What are your parents’ jobs?  [staff to categorise & code only the highest]  •No parents  •Not employed  •Agriculture  •Domestic services  •Unskilled manual  •Skilled manual  •Sales and services  •Clerical  •Professional/technical/managerial | We aligned the occupation  categories with the 2014 DHS,  adding domestic services |
| Income | What income band are you in?  •Less than 24,000 KSh/month  (288,000/yr, 10% Tax band)  •Between 24,000 - 32,333 KSh/  month (288,000 - 100,000/yr,  25% Tax band)  •More than 32,333 KSh/month  (388,000/yr, 30% Tax band) | N/A | We removed the question on food adequacy as we felt it was not likely to render robust information. We also dropped the subjective  question on income adequacy due to concerns about face validity. We replaced these income questions with a more direct item on income categories, based on the Kenya Revenue Authority tax bands |
| Housing | What is your floor made of in your house?  •Cement  •Other  Do you have a source of water within your compound?  • Yes  • No  Does your household have electricity, solar, or a generator?  •Yes  •No  What type of toilet facility do members of your households usually use?  • Own toilet/latrine  •Communal toilet/latrine  •None (bush/field) | What is your floor made of in your house?  •Cement  •Other  Do you have a source of water within your compound?  • Yes  • No  Does your household have electricity, solar, or a generator?  •Yes  •No  What type of toilet facility do members of your households usually use?  • Own toilet/latrine  •Communal toilet/latrine  •None (bush/field) | We switched from ‘earth, sand  or dung’ to ‘cement’. This is the  reciprocal question and is faster to ask.  We switched from ‘do you have water piped into your own house or yard?’ to ‘do you have a source of water within your compound’ because some rich people use boreholes  We revised the wording of the toilet question changed to add greater clarity  All options are aligned with the 2014 DHS |
| Assets | Do you own a smartphone?  •Yes  •No  Does your household own a:  •Bicycle  •Motorcycle/scooter  •Car or truck  •None  •Other | Does your household own a smart phone (with a touch screen)?  •Yes  •No  Does your household own a:  •Bicycle  •Motorcycle/scooter  •Car or truck  •None  •Other | We noted that smartphone ownership is so prevalent that it is only a sensible proxy for wealth in rural areas |

## Supplementary table 5: stratum specific effect estimates of association between attendance and age and gender

| **Strata** | **Category** | **Unadjusted OR** | **p-value** |
| --- | --- | --- | --- |
| 18-24 years | Female | Ref |  |
|  | Male | 0.43 (0.23-0.81) | 0.008 |
| 25-34 years | Female | Ref |  |
|  | Male | 0.57 (0.38-0.85) | 0.005 |
| 35-44 years | Female | Ref |  |
|  | Male | 0.55 (0.39-0.77) | 0.001 |
| 45-54 years | Female | Ref |  |
|  | Male | 0.58 (0.45-0.74) | <0.001 |
| 55-64 years | Female | Ref |  |
|  | Male | 0.88 (0.66-1.17) | 0.37 |
| 65+ years | Female | Ref |  |
|  | Male | 0.86 (0.64-1.15) | 0.305 |
| Women | 18-24y | 0.44 (0.31-0.62) |  |
|  | 25-34y | 0.44 (0.34-0.57) |  |
|  | 35-44y | 0.56 (0.44-0.70) |  |
|  | 45-54y | Ref |  |
|  | 55-64y | 1.08 (0.85-1.37) |  |
|  | 65+y | 1.46 (1.15-1.86) | <0.001 |
| Men | 18-24y | 0.33 (0.18-0.59) |  |
|  | 25-34y | 0.44 (0.30-0.65) |  |
|  | 35-44y | 0.53 (0.37-0.75) |  |
|  | 45-54y | Ref |  |
|  | 55-64y | 1.64 (1.22-2.22) |  |
|  | 65+y | 2.17 (1.61-2.94) | <0.001 |

Note: The p-value for the interaction term was 0.048

## Supplementary table 6: Regression with additional adjustment for eye condition


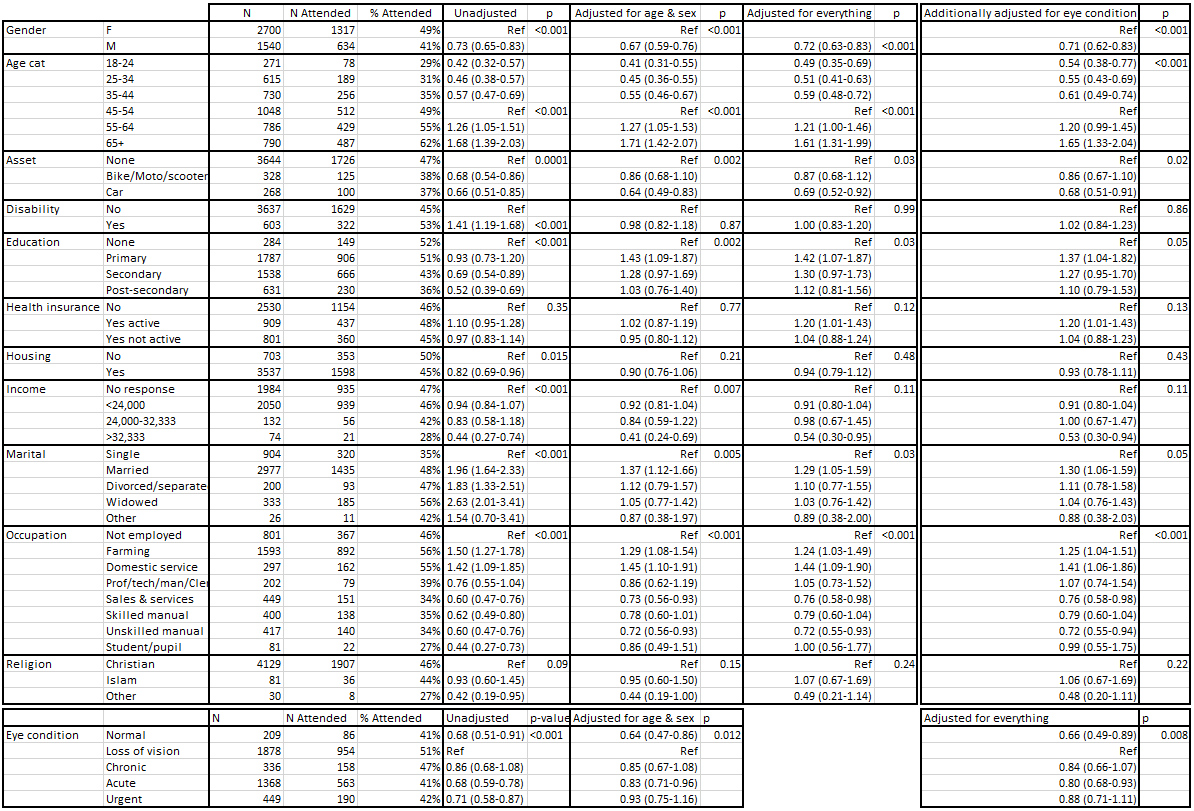

Supplement: Supplementary file 1 — Supplementary Material 1 [file 12939_2024_2244_MOESM1_ESM.docx]
